# Experimentally Derived Feasibility of Optical Camera Communications under Turbulence and Fog Conditions

**DOI:** 10.3390/s20030757

**Published:** 2020-01-30

**Authors:** Vicente Matus, Elizabeth Eso, Shivani Rajendra Teli, Rafael Perez-Jimenez, Stanislav Zvanovec

**Affiliations:** 1 Institute for Technological Development and Innovation in Communications, Universidad de Las Palmas de Gran Canaria, 35001 Las Palmas, Spain; rafael.perez@ulpgc.es; 2 Optical Communications Research Group, Northumbria University, Newcastle-upon-Tyne NE1 7RU, UK; elizabeth.eso@northumbria.ac.uk; 3 Department of Electromagnetic Field, Faculty of Electrical Engineering, Czech Technical University in Prague, Technicka, 16627 Prague, Czech Republic; telishiv@fel.cvut.cz (S.R.T.); xzvanove@fel.cvut.cz (S.Z.)

**Keywords:** optical camera communications (OCC), CMOS image sensor, rolling shutter, fog attenuation, heat-induced turbulence, meteorological visibility, refractive index structure parameter

## Abstract

Optical camera communications (OCC) research field has grown recently, aided by ubiquitous digital cameras; however, atmospheric conditions can restrict their feasibility in outdoor scenarios. In this work, we studied an experimental OCC system under environmental phenomena emulated in a laboratory chamber. We found that the heat-induced turbulence does not affect our system significantly, while the attenuation caused by fog does decrease the signal quality. For this reason, a novel strategy is proposed, using the camera’s built-in amplifier to overcome the optical power loss and to decrease the quantization noise induced by the analog-digital converter of the camera. The signal quality has been evaluated using the Pearson’s correlation coefficient with respect to a reference template signal, along with the signal-to-noise ratio that has been empirically evaluated. The amplification mechanism introduced allows our system to receive the OCC signal under heavy fog by gradually increasing the camera gain up to 16 dB, for meteorological visibility values down to 10 m, with a correlation coefficient of 0.9 with respect to clear conditions.

## 1. Introduction

Digital cameras are ubiquitous consumer electronics and are being explored to deliver extra capabilities beyond traditional photography and video. A new optical communication technique using cameras as receivers has been studied in the IEEE 802.15 SG7a within the framework of optical wireless communications and considered as a candidate of IEEE 802.15.7r1, which is called Optical Camera Communication (OCC). OCC has been investigated as one of the Visible Light Communication (VLC) schemes [1]. OCC implemented within internet of things (IoT) environments provides multiple functionalities of vision, data communications, localization and motion detection (MD) [2,3] used in various IoT-based network applications including device-to-device communications [4], mobile atto-cells [5], vehicular communications [6,7,8], and smart cities, offices, and homes (SCOH) [9].

The majority of new generation smart devices have built-in Complementary Metal-Oxide-Semiconductor (CMOS) image sensors, providing the ability to capture photos and videos [10,11]. The strategy behind using a CMOS camera for OCC is that the image sensor performs an acquisition mechanism known as Rolling Shutter (RS), in which it sequentially integrates light on rows of pixels [12] starting the scanning of each line with a delay with respect to the previous one. In other words, the timings of the line-wise scanning make the imaging sensor to capture different windows of time of the optical signal coming from a Light Emitting Diode (LED) transmitter ($T_{x}$). Then, each line of the image can hold a distinct portion of information.

The use of LEDs available in SCOH’s lighting infrastructures, along with optical receivers, for making VLC systems is particularly challenging in outdoor environments. The potential applications of OCC in these scenarios are related to the creation and improvement of communication networks for the vehicular and pedestrian infrastructures [13], where a large number of LED lights and CMOS cameras can be found. The desirable distance coverage of the different services that can take advantage of OCC ranges from a few meters for hand-held receiver devices based on smartphones, and tens of meters for vehicular networks that support Intelligent Transportation Systems (ITS). The achievable link distance in OCC depends partly on the signal-to-noise ratio (SNR) at the receiver, which in turn depends on the transmitted power, the attenuation caused by the channel, the optical lens array of the camera and various sources of noise and interference. In the case of RS-based systems, the maximum link distance is also restricted by the number of lines of pixels covered by the transmitter. For this, the geometry of the transmitting surface, as well as the image forming lens array configuration, determine the image area in pixels [14]. The modulation and packet scheme may have an impact on the maximum link distance if the image frames must contain a number of visible symbols for demodulation. Depending on the case of application, the LED and camera-based transceivers can either have static or mobile positions and orientations, making mobility support essential, which relies on the effective detection of the pixels that have an SNR level suitable for demodulation.

The vehicular VLC (VVLC) are a significant application case with challenging conditions of relative position and motion between nodes. An analysis based on a comparison of VVLC with radio frequency (RF) vehicle-to-vehicle (V2V) links in terms of channel time variation was proposed in [15]. It was shown that the VVLC links have much slower channel time variation as compared to RF V2V links. On the other hand, the VVLC investigation in [16] obtained link duration for VVLC between neighboring vehicles are more than 5 s while in certain other cases the average link duration can be up to 15 s. The safety regulations in [17,18] provide the speed limits and inter-vehicle distance in different weather conditions for the estimation of the desired distance of coverage. Table 1 shows the speed limit based on the European Commission regarding mobility and transport standards, which may vary slightly from one European country to the other. The inter-vehicle distances outlined have been calculated based on the 2 s driving rule for good to bad weather conditions, according to the Government of Ireland, which recommends that a driver maintains a minimum of two seconds apart form the leading vehicle for good weather conditions, which is doubled to four seconds in bad weather.

The performance of intensity-modulation and direct-detection method employed by LED-to-Photodiode (PD) VLC [9,19], is highly restricted by external light sources such as sunlight, public lighting, and signaling. Moreover, weather conditions, such as the presence of fog, or high temperatures, cause substantial optical distortions [20]. Addressing these challenges, authors in [21] derived a path-loss model for PD-to-LED VLC using Mie’s theory and simulating rain and fog conditions in a vehicular VLC setting. They determined the maximum achievable distances as a function of the desired bit-error-ratio (BER) using pulse amplitude modulation (PAM). They found that, for a 32-PAM system, the maximum distance achievable for the desired BER of $10^{-6}$ is reduced from 72.21 m in clear weather, to 69.13 m in rainy conditions, and 52.85 m and 25.93 m in foggy conditions of different densities. The same Mie’s theory is also used in [22] to evaluate a PD-based VLC link under maritime fog conditions. Scattering and phase functions are derived, as well as the spectrum of the attenuation of optical signals for different distances. In [23], the authors experimented with a LED-to-PD VLC link of 1 m distance based on a single 1 W red LED and multiple PDs attached to a Fresnel lens under dense fog conditions in a laboratory chamber. The lens allows them to maintain a 25 dB signal-to-noise ratio (SNR) varying the optical gain it provides to compensate the attenuation due to the fog presence.

Atmospheric turbulence, and oceanic turbulence in the case of Underwater Wireless Optical Communication (UWOC), has been extensively studied. Guo et al. introduced the traditional lognormal model into a simulated VLC link for ITS [24]. The authors proved that VLC wavelengths in ITS performed worse than longer ones (e.g., 1550 nm), which is straightforward, taking into account that the turbulence measured by Rytov’s variance has a dependence on the wavelength. In the case of UWOC, in which the use of visible-range wavelengths is mandatory due to the water absorption spectrum, Kolmogorov’s turbulence spectrum is substituted by Nikishov’s [25]. This turbulence spectrum fits better with the experimental measurements since it takes into account not only temperature but salinity variations.

Although the impact of turbulence has been characterized for classical optical detectors, its effect on OCC systems has not been adequately addressed yet. Works addressing channel characterization in outdoor OCC links [20] are still scarce compared to the amount of research on PD-based VLC. In the previous work [26], we evaluated the feasibility of a global shutter-based OCC link under fog conditions by the success rate of bits of vehicular link experimentally tested with a red brake light and a digital reflex camera. For a modulation index of 75%, the system showed high reliability under dense fog conditions up to a meteorological visibility of 20 m.

The contribution of this paper is to experimentally derive the feasibility of OCC in emulated outdoor conditions of fog and heat-induced turbulence using commercially available LEDs and cameras. This work is the first to report an experimental investigation on the effects of such conditions on an RS-based system. The experiments carried out for this work were done using a laboratory chamber, and the conditions emulated were of heat-induced turbulence and the presence of fog in the air. The refractive index structure parameter ($C_{n}^{2}$) [27] is used to estimate the level of turbulence and the meteorological visibility ($V_{M}$) as a measure of the level of fog. The fog experiments are especially relevant because we utilize the camera’s built-in amplifier to overcome the fog attenuation and mitigate the relative contribution of the quantization noise induced by the analog-to-digital conversion stage, ensuring an improvement of the signal quality without increasing the exposure time, and, thus, keeping a high bandwidth.

This paper is structured as follows. Section 2 describes the used methodology, including the channel modeling, the model for the meteorological phenomena studied, and it presents the experimental design. Section 3 presents the experimental setup, describing the laboratory chamber and the OCC system employed. Section 4 shows the obtained results for heat-induced turbulence and fog experiments and performs an in-depth discussion. Finally, conclusions are drawn in Section 5.

## 2. Methodology

In this section, we describe the relevant processes involved in the CMOS camera mechanism of acquisition in RS-based OCC employed by our system and derive the analytical tools used for the evaluation of its performance in the experimental setting.

### 2.1. Channel Modelling

**Figure 1:**
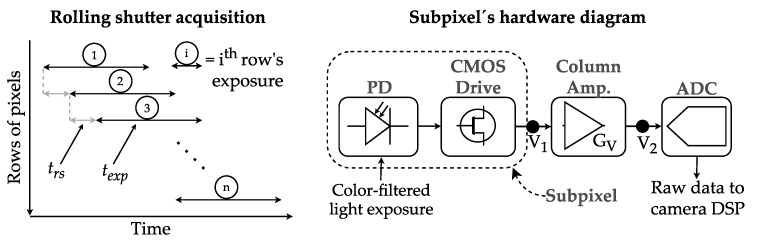
Typical configuration of Complementary Metal-Oxide-Semiconductor (CMOS) camera sub-pixels.

In CMOS image sensors, the red-green-blue (RGB) light from a Bayer filter impinges the subpixels. These entities are integrated by PDs and their driving circuit and are grouped by rows connected in parallel to amplifiers and analog/digital converter (ADC) units that are shared by columns. The output of these hardware blocks are image matrices that are sent to the camera digital signal processor (DSP), where data is compressed and delivered to the user as a media file. The sensor performs RS acquisition, in which the start and end of the exposure of each row of pixels are determined by the circuit’s fixed row-shift time ($t_{rs}$) and the software-defined exposure time ($t_{exp}$) [28]. The time parameters and circuitry mentioned are shown in Figure 1. Since $t_{rs}$ is fixed, in order to increase the data rate, $t_{exp}$ must be set as low as possible to make the sensor capture the highest diversity of states of the transmitter within each frame. The received power $P_{Rx}\left(t\right)$ at a camera coming from a Lambertian light source of order *m* and transmitted power $P_{Tx}\left(t\right)$ can be expressed as

(1) $$P_{Rx}\left(t\right)=P_{Tx}\left(t\right)\cdot \frac{m+1}{2\pi }\cdot \cos^{m}\theta \frac{A_{lens}\cos\Psi }{d^{2}},$$

where θ and Ψ are the emission and incident angles, respectively, $A_{lens}$ is the area of the camera’s external lens, and *d* is the link span. From the RS mechanism shown in Figure 1, we can express the energy $E_{i}$ captured by the $i^{th}$ row as

(2) $$E_{i}=\int_{i\cdot t_{rs}}^{i\cdot t_{rs}+t_{exp}}\frac{P_{Rx}\left(t\right)}{\sum_{j}^{v}\sum_{k}^{h}M_{j,k}}dt,$$

where *h* (columns), *v* (rows) are the dimensions of the image sensor, and $M_{\left[v\times h\right]}$ is the mask of pixels where the source shape is projected. From the integral limits, it can be derived that the bandwidth of the $R_{x}$ system decreases with the augment of the exposure time. In other words, the longer is $t_{exp}$, the more lines are simultaneously exposed, and the received signal is integrated in longer and less diverse time windows. For this reason, frames in OCC have to be acquired within short periods.

Note that low values of $t_{exp}$, along with the attenuation factor in outdoor channels caused by the presence of particles such as fog or by the light refraction by turbulence can result in $E_{i}$ lower than the sensor’s lowest threshold of detection. For overcoming this, we can take advantage of the amplifying stage in the subpixel circuitry shown in Figure 1. The voltage-ratio gain $G_{V}$ of the column amplifier block behaves as

(3) $$G_{V}\left(dB\right)=20\log_{10}\left(V_{2}/V_{1}\right),$$

where $V_{1}$ is the voltage obtained from the pixel integration of light during exposure, and $V_{2}$ is the voltage value that is sampled by the ADC. In the case of the IMX219 sensor and of other CMOS sensors with the architecture shown in Figure 1, a software-defined analog gain configuration can set the value of $G_{V}$ for each capture. The typical values of $G_{V}$ range from 0 dB to 20.6 dB, as shown in [29].

The column gain $G_{V}$ of the CMOS sensor amplifies the received signal $P_{Rx}$ and all the noises up to the ADC. This includes the shot noise at the PD, and the thermal noises of the circuits, which can be modeled as random variables of Normal distributions with variances $\sigma_{sh}^{2}$, and $\sigma_{th}^{2}$, as:

(4) $$\sigma_{sh}^{2}=2q_{e}\left(i_{pd}\left(x,y,c\right)+i_{d}+i_{bg}\right)B,$$

(5) $$\sigma_{th}^{2}=\frac{4k_{B}T_{n}B}{G_{V}},$$

where $k_{B}$ is the Boltzmann’s constant, $T_{n}$ is noise temperature, $B=1/t_{rs}$ is the bandwidth, $q_{e}$ is the electron charge, $i_{d}$ is the dark current of the camera’s pixels, and $i_{pd}\left(x,y,c\right)$ is the PD current at pixel (x,y) in the color band c∈{R,G,B}. This current is determined by the emitted spectrum of the light source, the corresponding Bayer filter, and the substrate’s responsivity. Finally, $i_{bg}$ models the contribution of the background illumination level to the shot noise. Nonetheless, since reduced exposure times are generally used, the contribution of $i_{bg}$ can be neglected.

The signal is then sampled by the ADC, introducing quantization noise ($\sigma_{adc}^{2}$), that is usually modeled as a zero-mean random normal contribution whose variance depends on the resolution of the converter. This results in the SNR, which is referred at the DSP’s input as:

(6) $$SNR\approx \frac{G_{V}\cdot i_{pd}^{2}\left(x,y,c\right)}{G_{V}\left(\sigma_{th}^{2}+\sigma_{sh}^{2}\right)+\sigma_{adc}^{2}}.$$

Considering the SNR as a function of $G_{V}$, it can be observed that it has an increasing behaviour with an upper asymptote given by $i_{pd}^{2}\left(x,y,c\right)/\left(\sigma_{th}^{2}+\sigma_{sh}^{2}\right)$. Especially in the cases when the signal entering the ADC is weak, e.g., as in high attenuation scenarios such as in the presence of dense fog, the relative loss due to quantization noise can be minimized by increasing the column amplification. In other words, the SNR can be optimized by the camera analog gain, unless the ADC is saturated.

Our system employs an On-off keying (OOK) modulation for each of the color bands with a fixed data input that is used as a beacon signal. For bit error ratio (BER) derivation, let us assume the system now works with a random data input of $p_{0}=p_{1}=0.5$ as the probabilities of value 0 and 1, respectively. The Maximum Likelihood Estimator (MLE) threshold $\mu_{mle}$ at the detection stage of the OOK demodulation is given by

(7) $$\mu_{mle}=\left(\mu_{0}+\mu_{1}\right)/2,$$

where $\mu_{0}$ and $\mu_{1}$ are the expected values of the received signal for the cases of transmitted signal equal to bits 0 and 1, respectively. If the receiver’s DSP applied a digital gain $k_{d}$, the resulting MLE threshold would be $\tilde{\mu_{mle}}=k_{d}\left(\mu_{0}+\mu_{1}\right)/2$. In this case, if $\mu_{1}<2^{n_{bit}}$, where $n_{bit}$ is the bit depth, and $2^{n_{bit}}$ is the maximum digital value of the signal coming from the ADC, the BER would tend to the worst case of a coin flip (error probability equal to 0.5).

### 2.2. Meteorological Phenomena

The presence of fog particles and turbulence in the air are known as relevant sources of signal distortion in outdoor optical systems. These conditions can be emulated in a laboratory chamber, and well-known parameters can estimate their degree, as explained in the following derivations.

Beer’s law [30] can describe the attenuation of propagating optical signals caused by fog. Generally, in optical systems, visibility $V_{M}$ in km is used to characterize fog attenuation ($A_{f}$). Using the Mie’s scattering model [31], $A_{f}$ can be related to $V_{M}$ as:

(8) $$A_{f}=\frac{3.91}{V_{M}}{\left(\frac{\lambda }{550}\right)}^{-q},$$

where λ denotes wavelength in nm and parameter *q* is the distribution size of scattering particles given by Kim’s model [32], which is in the short range of visibility (<0.5 km) considered equal to zero. Thus, $V_{M}$ is given by:

(9) $$V_{M}=\frac{3.91}{A_{f}}.$$

The channel coefficient for fog $h_{f}$ can be determined by applying Beer’s law describing light scattering and absorption in a medium as:

(10) $$h_{f}=e^{-A_{f}d}.$$

Consequently, the average received optical power for the LOS link at the $R_{x}$ under fog is expressed as:

(11) $$P_{Rxf}\left(t\right)=P_{Rx}\left(t\right)h_{f}+n\left(t\right),$$

where n(t) denotes the addition of noises associated with $\sigma_{th}^{2}$ and $\sigma_{sh}^{2}$.

The coefficient $h_{f}$ depends on the value of the product of fog-attenuation and distance ($A_{f}\cdot d$), which is known as the optical density of the link. This variable can have the same value for different combinations of fog level and link span, allowing to infer the influence of both variables varying only one of them.

The heat-induced turbulence of air results from variations in temperature and pressure of the atmosphere along the path of transmission. Consequently, this leads to variations of the refractive index of the air, resulting in amplitude and phase fluctuations of the propagating optical beam [33]. For describing the strength of atmospheric turbulence, the parameter most commonly used is the refractive index structure parameter ($C_{n}^{2}$) (in units of m${}^{-2/3}$) [34,35], given by:

(12) $$C_{n}^{2}={\left(79\cdot 10^{-6}\frac{P}{T^{2}}\right)}^{2}\cdot C_{T}^{2}$$

where *T* represents temperature in Kelvin, *P* is pressure in millibar, $C_{T}^{2}$ is the temperature structure parameter which is related to the universal 2/3 power law of temperature variations [35] given by:

(13) $$D_{T}=\langle {\left(T_{1}-T_{2}\right)}^{2}\rangle =\left\{\begin{array}{cc} C_{T}^{2}\cdot L_{P}^{2/3} & l_{0}\ll L_{P}\ll L_{0}\\ C_{T}^{2}\cdot l_{0}^{-4/3}\cdot L_{P}^{2} & 0\ll L_{P}\ll l_{0} \end{array}\right.,$$

where $|T_{1}-T_{2}|$ is the temperature difference between two points separated by distance $L_{P}$, while the outer and inner scales of the small temperature variations are denoted by $L_{0}$ and $l_{0}$, respectively.

### 2.3. Experimental Design

**Figure 2:**
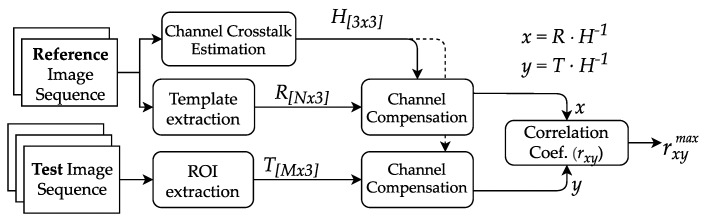
Flow diagram of the offline processing of data captured by cameras.

For the OCC system to be tested under emulated meteorological phenomena, the following conditions were considered. The signal transmitted by the VLC lamp was chosen to be a repetitive beacon, formed by a sequence of on-off pulses of each of the RGB channels, and followed by a black (off state) pulse denoted as K, then, the beacon was arbitrarily set to the following: G-R-B-K. The K pulse allows measuring the dark intensity in the pixels that cover the lamp image, while the pure color pulses allow to estimate the inter-channel cross-talk between the LED RGB colors and the RGB subpixels of the camera, as explained in our previous work [36]. The $R_{x}$ camera equipment was configured to take captures with fixed $t_{exp}$ and different $G_{V}$ sequentially. After taking reference measurements, the atmospheric conditions were emulated while the beacon transmission and capture processes were sustained. The reference and test image sequences are processed through the stages shown in Figure 2, including the extraction of relevant pixels area in the picture, the estimation and enhancing of inter-channel cross-talk, and finally, the computation correlation between the signals obtained in clear conditions and under emulated weather conditions.

The extraction of the relevant group of pixels in OCC image frames, known as Region of Interest (ROI) detection, consists of locating the projection of the source in the image. In this case, we first manually locate and extract the ROI from the reference sequence. Then, since the test group is taken with the same alignment, the ROI stays fixed. Thus, the same coordinates of it are re-utilized. The pixels containing data are then averaged by row, giving the three-channel (RGB) signal $T_{\left[M\times 3\right]}$, where *N* is the number of rows of the ROI. From the reference ROI, a template of one G-R-B-K beacon signal is saved as $R_{\left[N\times 3\right]}$, where *M* is the number of rows used by one beacon in the RS acquisition.

As shown in previous work [36], the inter-channel cross-talk (ICCT), which is caused by the mismatch between the LEDs and the camera’s Bayer filter spectra, is estimated from clear frames and then compensated in all datasets. We separately analyze R, G, and B pulses from the beacon signal. A matrix $H_{\left[3\times 3\right]}$ is obtained by averaging the contribution of each pure-LED pulse at the three RGB subpixels. In other words, a component $h_{ij}$ from $H_{\left[3\times 3\right]}$ is the average measure from the $j^{th}$ subpixel when the $i^{th}$ LED is illuminating it, where i,j∈{R,G,B}. The inverse matrix $H_{\left[3\times 3\right]}^{-1}$ is used to clean all the datasets from ICCT found at this configuration. Finally, ICCT cleaned signals $x={\left(R\cdot H^{-1}\right)}_{\left[N\times 3\right]}$ and $y={\left(T\cdot H^{-1}\right)}_{\left[M\times 3\right]}$ are compared using the Pearson’s correlation coefficient $r_{xy}$, which is defined as:

(14) $$r_{xy}=\frac{\sum_{i=1}^{N}\left(x_{i}-\bar{x}\right)\left(y_{i}-\bar{y}\right)}{\sqrt{\sum_{i=1}^{N}{\left(x_{i}-\bar{x}\right)}^{2}}\sqrt{\sum_{i=1}^{N}{\left(y_{i}-\bar{y}\right)}^{2}}},$$

where $x_{i}$ are the reference sample points from *R*, of size *N*, $y_{i}$ are *N* consecutive samples of *T*, and $\bar{x},\bar{y}$ are the mean values. The correlation is calculated for all possible consecutive subsets $y_{j},y_{j+1},\ldots ,y_{j+N-1},\left(j+N-1\right)<M$ and the maximum value $r_{xy}^{max}$ is considered the similarity of the frame compared to the reference.

## 3. Experimental Setup

**Figure 3:**
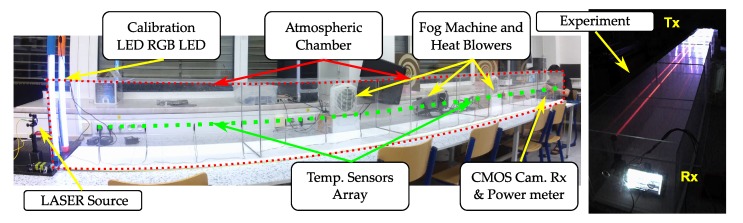
Photos of the laboratory setup utilized in the experiments.

**Figure 4:**
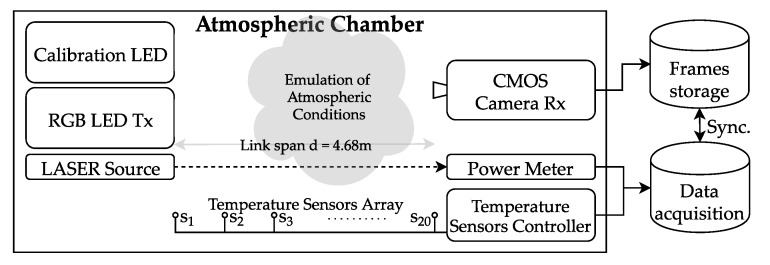
Block diagram of the experimental setup.

In this section, we describe the full setup of our experiments, which is shown in Figure 3, including the laboratory chamber used, the tools used for emulating hot and foggy weather conditions, the measurement devices used for estimating the levels of each condition, and the $T_{x}$ and $R_{x}$ devices that comprise the OCC link. The key experiment parameters are listed in Table 2 and the block diagram of the experimental setup is shown in Figure 4.

### 3.1. Laboratory Chamber

The atmospheric chamber set up for measurements in the facilities of the Czech Technical University in Prague [27] features two heater fans, and one Glycerine machine, that can blow hot air and fog into the chamber, respectively. For the characterization of turbulence and light scintillation in the chamber, an array of 20 temperature sensors were set up equidistantly. A laser source of 625 nm and 2 mW, and an optical power meter placed on each end of the chamber are used to measure the fog attenuation.

### 3.2. OCC System

The transmitter unit was built using strips of RGB LEDs connected to a microcontroller (model ATmega328p [37]) through a switching circuit based on transistors. The LED arrays were installed on aluminum rails with a white meth-acrylate diffuser. The circuitry makes the RGB channels to emit the beacon signal (idle state) repeatedly, or to send arbitrary data coming from a serial port (this feature was not used in this experiment). The chip time $t_{chip}$ or the pulse width is set by software in the microcontroller. In the case of the experiments, this parameter was set to 1/8400 s.

The receiver was made using an Element14 Raspberry Pi board with its official camera device PiCamera V2. The firmware allows to set $G_{V}$ from 1 to 16 dB and exposure time from 20 ns up to the time elapsed between frame captures, which in case of 30 fps video is approximately 33.3 ms. The fixed internal structure of the CMOS sensor (Sony IMX219) featured by the PiCamera is set to have a row-shift time $t_{rs}$ = 18.904 μs [29]. The exposure time was set to $t_{exp}$ = 60 μs.

Given the hardware configuration of our system in the laboratory, as shown in Figure 3, each of the image frames can contain up to 64 symbols. Since the modulation uses RGB channels, each symbol then is formed by 3 bits. The maximum throughput of this configuration at 30 fps is then 5.76 kbps.

## 4. Results

In this section, we show the results from the analysis of the images obtained from heat-turbulence and fog experiments carried out, as shown in Section 3. The maximum values of the correlation coefficient were computed between the ICCT-compensated reference image sequence and the images captured under different conditions, as explained in Section 2. The $r_{xy}^{max}$ values obtained are analyzed together with the experimental parameters set: $C_{n}^{2}$ in the case of heat-induced turbulence, and $V_{M}$, $G_{V}$, in the case of fog.

### 4.1. Heat-Turbulence Experiments

The heat-turbulence experiment’s reference image sequence was captured using the chamber heaters off at a stabilized laboratory temperature of 21.7 °C. Thus, the template signal extracted from these captures is the result of operating the system under a negligible level of turbulence. The remaining test image sequence was captured under the thermal influence of channel in two parts, one under a higher laboratory temperature of 32.3 °C, and a second part with the heaters of the chamber working at full power, setting another turbulence level. The $C_{n}^{2}$ parameter value is then calculated using the temperature sensors samples. The $r_{xy}^{max}$ values between the frames of the test image sequence and the template are calculated. With these values, we infer the influence of this phenomenon.

**Figure 5:**
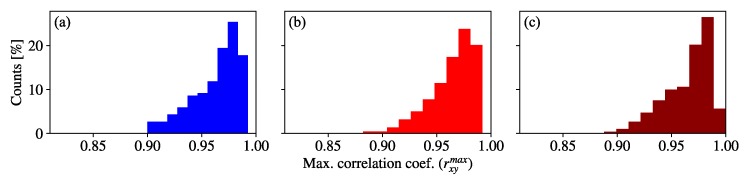
Distribution of maximum correlation coefficient values of image sequences taken (**a**) under a cool room temperature of 21.7 °C (no turbulence), (**b**) under a warm room temperature of 32.3 °C and with heaters off, and (**c**) with turbulence induced by the heaters.

The refractive index structure parameter values during the first part of the test image sequence capture ranged from $C_{n}^{2}=1.86\cdot 10^{-11}$ m${}^{-2/3}$ to $2.51\cdot 10^{-11}$ m${}^{-2/3}$ in high room temperature with the heaters off. In the second part, the range of turbulence increased to $4.69\cdot 10^{-11}$ m${}^{-2/3}\leq C_{n}^{2}\leq 7.13\cdot 10^{-11}$ m${}^{-2/3}$. The obtained $r_{xy}^{max}$ between the signals from each part of the experiment and the template are shown as histograms in Figure 5. To estimate the similarity between the $r_{xy}^{max}$ data from the reference and from each part of the test image sequence, a Kolmogórov-Smirnov (KS) statistical test was done, which consists of a non-parametric tool that estimates if two data sets are samples from the same distribution with a confidence *p*-value [38]. The result is that the first part of the test image sequence has p=0.81 confidence value of having the same distribution as the reference, and the second has p=0.83. It can be seen an almost negligible influence of turbulence on OCC systems.

The different ranges of turbulence analyzed presumably have the same distribution of $r_{xy}^{max}$ values, according to the KS statistical test, and also the vast amount of them meet that $r_{xy}^{max}>0.9$, which means that the experimental setup’s behavior is considerably similar to the reference, regardless of the turbulence ranges that were induced. This robustness of the system can be attributed to the short link distance and the big field of view of the camera. Both make the refraction effects unnoticeable in the received signal of our system.

### 4.2. Fog Experiments

For the fog emulation experiment, the reference image sequence was taken under clear air in the laboratory chamber while the optical power meter measured the power of the laser without fog attenuation. The test image sequence was taken while the chamber was arbitrarily supplied with fog from the Antari F-80Z, while the laser power was measured in synchronicity in order to label each image with the current $V_{M}$. The value of $G_{V}$ of the images was sequentially modified from 0 to 16 dB by steps of 1 dB during the test image sequence, while for the reference, it was set to zero as default.

**Figure 6:**
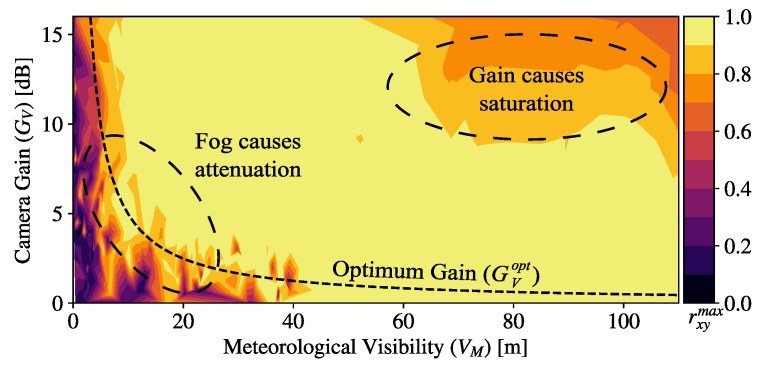
Maximum correlation between test and reference signals varying camera gain under emulated fog conditions of different values of meteorological visibility.

**Figure 7:**
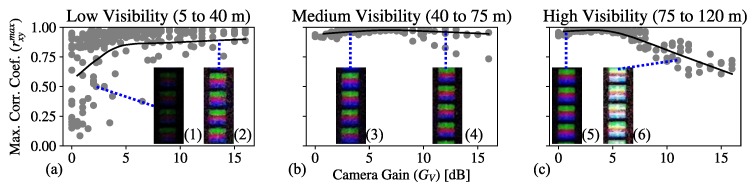
Maximum correlation data (gray dots) from fog-emulation experiments, separated by levels of (**a**) low, (**b**) medium, and (**c**) high visibility and their respective locally estimated scatterplot smoothing (LOESS) regression for s=0.5 (black curves). The area encircled in (**a**) is the region of image frames affected by the fog attenuation and in (**b**) by gain saturation. Insets are Region of Interest (ROI) extraction examples: (1) for low visibility and low gain, (2) low visibility and high gain, (3) medium visibility and low gain, (4) medium visibility and high gain, (5) high visibility and low gain, and (6) high visibility and high gain.

The $r_{xy}^{max}$ values obtained for the test images sequence varying $G_{V}$ and $V_{M}$ are shown as a contour plot in Figure 6. The high correlation area ($r_{xy}^{max}>0.9$) determines three important regions (highlighted in Figure 6 by dashed circles). For the high values of visibility, the signal coming from the transmitter is not affected by the fog attenuation and is received with the highest power. Then, the increase of gain causes saturation of the ADC, affecting the correlation. In the low visibility region, the presence of dense fog attenuates the received signal and lowers the correlation. It can be seen that, in this low-visibility region, the increase of gain gives a high correlation, meaning that the camera amplifier compensates the attenuation from fog. The region in between, around 50 m visibility, shows high values of correlation regardless of the variations of gain. The three regions described are shown in Figure 7, and a non-parametric locally estimated scatterplot smoothing (LOESS) regression [39] is performed with parameter span s=0.5 to show the trend of the data points. Examples of the ROI extraction from test images sequence are included to depict the effect of visibility and gain over the frames.

From the minimum gain values in the area of $r_{xy}^{max}>0.9$, an optimum gain curve $G_{V}^{opt}$ is derived providing that there is an inverse proportionality relationship between meteorological visibility and camera gain as follows:

(15) $$G_{V}^{opt}\left(V_{M}\right)=\frac{k_{v}}{V_{M}},$$

where $k_{v}$ is an empirical parameter. Using curve fitting, the value $k_{v}=0.0497$ dB·km was derived for our experimental setup.

In order to calculate the SNR from the empirical data obtained, we have considered that OOK modulation is used. The following approximation of the SNR has been derived (note the 1/2 factor due to OOK):

(16) $$SNR=\frac{1}{2}\frac{E^{2}\left[X_{ROI}\right]}{V\left[X_{ROI}\right]},$$

where $X_{ROI}$ comprises the samples of pixels that fall within the ROI mask $M_{\left[v\times h\right]}$ as described in Equation (2), which was determined from reference images and since the $T_{x}$ and $R_{x}$ are static it is the same for the whole experiment. E[·], and V[·] denote the statistical expected value and variance, respectively.

**Figure 8:**
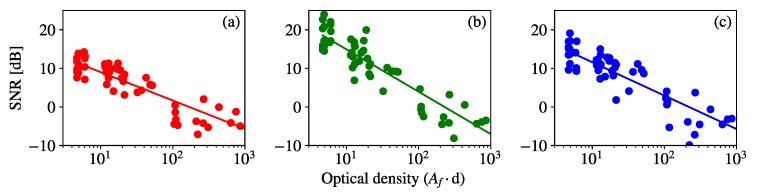
Empirical signal-to-noise ratio (SNR) values obtained from captures at $G_{V}$ = 11 dB plotted against optical density values at fixed link range *d* and with $A_{f}$ values emulated by the presence of fog. The plot in (**a**) corresponds to R channel, (**b**) to G channel, and (**c**) to B channel, and their respective fitted curves.

The empirical SNR definition was calculated for all the image sequences of the fog experiments. The results for the frames taken with $G_{V}$ = 11 dB are shown in Figure 8 for the three RGB channels. This value of gain was chosen because, as shown in Figure 6, the level $G_{V}$ = 11 dB is affected by the dense fog and also by the saturation. The SNR values in Figure 8 are plotted against optical density in logaritmic scale. They show that higher attenuation $A_{f}$ values, or alternatively, longer link spans, cause a decay of the SNR. Therefore, a curve fitting was carried out assuming that the SNR decays at a rate of α dB per decade of optical density, as follows:

(17) $$SNR\left(A_{f}d\right)=SNR\left(1\right)+\alpha \cdot \log\left(A_{f}d\right),$$

where SNR(1) is the estimated signal-to-noise ratio at unitary optical density.

**Figure 9:**
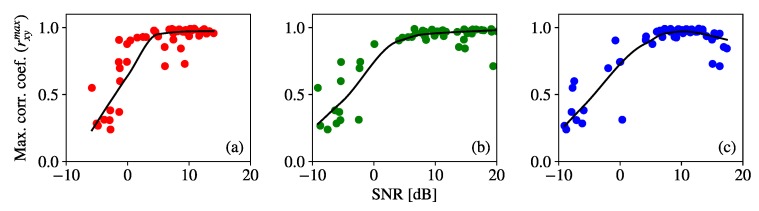
Values of $r_{xy}^{max}$ from test image sequence of the fog-emulation experiment plotted against empirical SNR. The values are for frames taken with $G_{V}$ = 11 dB. The scatter plot in (**a**) corresponds to R channel, (**b**) to G channel, and (**c**) to B channel, and the curves in black are their corresponding LOESS regression for span value s= 0.7.

The SNR values obtained from the image sequences were also evaluated on their influence over $r_{xy}^{max}$, as shown in Figure 9. A LOESS regression also shows the trend of the scatterplots in the figure, and it can be seen that $r_{xy}^{max}$ increases with the SNR, except for the highest SNR values in the blue channel, which are affected by saturation of the ADC. It can also be seen that SNR values higher than 5 dB make $r_{xy}^{max}>0.9$ for most of the samples. From this, it can be concluded that $r_{xy}^{max}$ is a valid metric for the quality of the signal in OCC, although SNR is more robust.

The results obtained in this experiment show that the fog attenuation can make the power of the optical signal weaken down to the point that the noise induced by the ADC considerably affects the SNR. In other words, the conversion to digital corrupts the weak optical signal from dense fog conditions or long link spans. In these cases, the column amplifier of the camera is crucial to keep a high amplitude input at the ADC and reduce the effect of quantization.

## 5. Conclusions

In this paper, we presented an experimental study of the influence of two kinds of atmospheric conditions over an RS-based OCC link: the heat-induced turbulence due to random fluctuations of the refractive index of the air along the path, and the attenuation caused by the presence of fog particles in the air. The image sequences captured under the two different conditions were compared to a reference sequence of images taken under clear conditions. For this, we used the maximum value of Pearson’s correlation coefficient $r_{xy}^{max}$ to determine their similarity. We have also evaluated the signal quality by the empirical SNR obtained from the image frames and showed its relationship with $r_{xy}^{max}$ and its dependence on the product between fog attenuation and link span, known as the optical density. The most important findings in this work are, first, that the turbulence levels emulated do not affect the signal quality considerably. For the fog experiments, we have derived an expression for the theoretical SNR as a function of the analog camera gain, showing that a CMOS camera-based OCC system can improve the SNR by using the column amplifier. In the fog experiments, the correlation $r_{xy}^{max}$ was impaired in two different cases: for high values of $V_{M}$, when the gain is increased, the correlation drops because of the saturation of the signal, and, for low visibility, the attenuation caused by the fog impairs the similarity to the reference when the gain is low, because of the loss due to quantization noise at the ADC. It was found for the latter case that by increasing the gain of the camera, the attenuation can be compensated, allowing the OCC link to receive signal with a $r_{xy}^{max}>0.9$ for $V_{M}$ values down to 10 m. Our findings show that there is an inverse proportionality relationship between the optimum camera gain and the visibility, and that the empirical SNR decays at a rate α with the optical density. This utilization of the CMOS camera’s built-in amplifier opens a new possibility for OCC systems, extending the control strategy, and allowing to keep low exposure times and, thus, a high bandwidth, even in dense fog scenarios.

## Figures and Tables

**Table 1:** Inter-vehicle distances based on the weather condition based on regulations in [17,18].

	Speed Limits [km/h]		Inter-Vehicle Distance [m]	
Weather condition	Motor ways	Rural roads	Motor ways	Rural roads
Good weather	120–130	80–90	67–72	44–50
Bad weather ($V_{M}$ = 50 m)	50	50	56	56

**Table 2:** Experiment key parameters.

Parameter	Value
Transmitter	
Device	12 V DC RGB LED strips (108 × 5050 SMD chips)
Front-end device	Microcontroller Atmel ATMega328p [37]
Idle power [W]	4.76
Dominant wavelengths [nm]	630 (Red), 530 (Green), 475 (Blue)
$T_{chip}$ [s]	1/8400
Receiver	
Camera	Picamera V2 module (Sony IMX219)
Resolution	3280×2464 px
$t_{exp}$ [μs]	60
Gain ($G_{V}$) [dB]	0, 1, …, 16
Frame rate [fps]	30
Laboratory chamber	
Dimensions [m]	4.910×0.378×0.368
Temperature sensors	20 × Papouch Corp. TQS3-E (range: −55 to +125 °C×0.1 °C)
LASER source	Thorlabs HLS635 (635 nm) F810APC
Optical power meter	Thorlabs PM100D S120C
Heat blowers	2 × Sencor SFH7010, 2000 W
Fog machine	Antari F-80Z, 700 W
